# Protective effect and pharmacokinetics of dihydromyricetin nanoparticles on oxidative damage of myocardium

**DOI:** 10.1371/journal.pone.0301036

**Published:** 2024-04-16

**Authors:** Lixin Du, Huiling Lu, Yifei Xiao, Zhihua Guo, Ya Li

**Affiliations:** 1 School of Pharmacy, Hunan University of Chinese Medicine, Changsha, China; 2 School of Chinese Medicine, Hunan University of Chinese Medicine, Changsha, China; Brandeis University, UNITED STATES

## Abstract

**Purpose:**

This study aims to investigate the protective mechanism of dihydromyricetin PLGA nanoparticles (DMY-PLGA NPs) against myocardial ischemia-reperfusion injury (MIRI) in vitro and the improvement of oral bioavailability in vivo.

**Methods:**

DMY-PLGA NPs was prepared and characterized by emulsifying solvent volatilization, and the oxidative stress model of rat H9c2 cardiomyocyte induced by H_2_O_2_ was established. After administration, cell survival rate, lactate dehydrogenase (LDH), malondialdehyde (MDA) and superoxide dismutase (SOD) were detected, and the expressions of PGC1α and PPARα were detected by western blot (WB). At the same time, the pharmacokinetics in rats were studied to explore the improvement of bioavailability.

**Results:**

DMY-PLGA NPs can significantly increase cell survival rate, decrease LDH and MDA content, increase SOD content and PGC1α、PPARα protein expression. Compared with DMY, the peak time of DMY-PLGA NPs was extended (*P*<0.1), and the bioavailability was increased by 2.04 times.

**Conclusion:**

DMY-PLGA NPs has a significant protective effect on H9c2 cardiomyocytes, which promotes the absorption of DMY and effectively improves bioavailability.

## 1 Introduction

Cardiovascular disease (CDV) is the collective name of heart and blood vessel diseases, which is the main disease that causes human death worldwide, and is a serious threat to human health. CDV includes a series of pathological conditions affecting the heart and blood vessels, and myocardial ischemia is one of its common forms. Myocardial ischemia-reperfusion injury (MIRI) refers to a pathological process in which the ischemic myocardium is restored to normal perfusion but the tissue damage is progressively aggravated when the coronary artery is re-routed within a certain period of time after obstruction [1]. As one of the main pathophysiological bases of heart diseases such as myocardial infarction, MIRI is highly likely to cause the destruction of myocardial cell membranes, resulting in death and loss of function of myocardial cells, and in severe cases, arrhythmia or heart failure [2]. The mechanism of MIRI involves many theories such as neutrophil infiltration, calcium overload and energy metabolism disorder, among which the theory of oxidative stress is the current hot spot. Oxidative stress refers to the excessive production of reactive oxygen species in the body or the decrease of antioxidant capacity, which causes a large amount of reactive oxygen species to accumulate and damage the body. Reactive oxygen species can damage cardiomyocytes through various ways, such as directly attacking cells to cause cell necrosis or activating redox signaling pathway to induce cell apoptosis [3, 4]. Therefore, it is of great significance to find an effective treatment for MIRI.

Dihydromyricetin (DMY), with its molecular formula of C_15_H_12_O_8_, mainly exists in the genus vitis of the vitis family. It is a flavonoid with significant physiological activity, and has functions such as antioxidant, antialcoholic liver, anti-tumor, kidney protection, cardiovascular protection, lowering blood sugar and blood pressure, and antibacterial and anti-inflammatory [5, 6]. In recent years, there have been numerous studies on the treatment of cardiovascular diseases by DMY. A large number of studies [7–9] have shown that DMY can inhibit endothelial damage, participate in lipid metabolism and affect the process of atherosclerosis, regulate mitochondrial metabolism and functional changes of cardiomyocytes, and inhibit oxidative stress-induced apoptosis in the treatment of MIRI. However, DMY is poor in water solubility, unstable to light and heat, and low in bioavailability [10], which greatly hinders its application and development.

Nanoparticle drug delivery system (NDDS) is a product of the application of nanotechnology in the field of medicine, which can increase the permeability of biofilm, increase the targeted distribution of drugs, improve the stability of drugs, prolong the action time of drugs in vivo and reduce toxicity and improve bioavailability [11, 12]. Among them, nanoparticles are widely used. Polylactic-glycolic acid copolymer (PLGA) is a nanoparticle carrier material approved by the FDA [13, 14], which is safe, non-toxic, has good biocompatibility and biodegradability, then its degradation products in human body can be excreted through metabolism. Many phytochemicals have been made into nanoparticles using PLGA as a carrier, such as curcumin [15], magnolol [16], andrographolide [17], etc. These substances are wrapped in PLGA nanoparticles or adsorb on the surface, which greatly prolongs the action time in the body.

In this study, DMY-PLGA NPs was prepared using PLGA as the carrier, and the shape characteristics of DMY-PLGA NPs were revealed through a series of physical characterization. In order to investigate the therapeutic effect of DMY-PLGA NPs on MIRI, we evaluated it in vivo and in vitro. H9c2 cardiomyocytes were treated with H_2_O_2_ to establish a model of oxidative stress in vitro, and the levels of related indexes and protein expression of oxidative stress, including LDH, MDA, SOD, PGC1α and PPARα were determined. Secondly, the ability of DMY-PLGA NPs to prolong drug cycle time was validated by pharmacokinetic studies in rats after oral administration.

## 2 Materials and methods

### 2.1 Materials

Dihydromyricetin and apigenin were purchased from China National Institute of Food and Drug Control (Nanjing, China). Ethyl acetate, phosphoric acid, methanol and acetone were purchased from Sinophosphate Chemical Reagent Co., LTD (Shanghai, China). Poloxam 188, PLGA (75:25, molecular weight: 30000) were purchased from Jinan Daigang Biotechnology Co., LTD (Jinan, China). Culture-medium, trypsin and fetal bovine serum were purchased from Wuhan Punosai Biological Co., LTD (Wuhan, China). CCK-8 kit was purchased from Beijing Boosen Biological Reagent Co., LTD (Beijing, China). LDH, MDA and SOD kit were purchased from Eloret Biotechnology Co., LTD (Wuhan, China).

Rat H9c2 myocardial cell line was purchased from Shanghai Cell Bank of Chinese Academy of Sciences, and 12 SPF-grade SD male rats were purchased from Hunan Lacke Jingda Laboratory Animal Co., LTD, certificate number: 430727221101958114. All animals are raised by the Animal Experimental Center of Hunan University of Chinese Medicine in accordance with the "Experimental Animal Care and Use Guide", examined by the Animal Ethics Committee of Hunan University of Chinese Medicine and meet the standards, ethics approval number: LLBH-202108110001.

### 2.2 Preparation of DMY-PLGA NPs

PLGA 20.54 mg and DMY 3.95 mg were accurately weighed and dissolved in 1 mL acetone to form organic phase by ultrasonic dissolution. 10 mL of 0.4% poloxam 188 aqueous solution was prepared as water phase. The water phase was placed in a magnetic stirrer, the rotational speed was adjusted to 1200 rpm, the organic phase was slowly injected into the water phase, then the rotational speed was adjusted to 600 rpm, and the mixture was stirred at room temperature until the acetone was completely volatilized. DMY-PLGA NPs was obtained after 10 min of ultrasound in the ultrasonic cell grinder.

### 2.3 Characterization of DMY-PLGA NPs

#### 2.3.1 Particle size and potential

Take appropriate samples in quartz colorimetric dishes and sample pools, the particle size, potential and dispersion index (PDI) of DMY-PLGA NPs were determined by using Malvern laser particle size analyzer.

#### 2.3.2 Transmission electron microscope (TEM)

DMY-PLGA NPs suspension 10 μL was dropped on the copper net, the excess liquid was absorbed, and the morphology was observed under transmission electron microscope after staining with 2% phosphotungstic acid for 30 s.

#### 2.3.3 Fourier transform infrared spectroscopy (FTIR)

DMY, blank nanoparticles without DMY, and DMY-PLGA NPs were detected under Fourier infrared spectrometer, and the scanning range was 4000∼400 cm^-1^.

#### 2.3.4 Encapsulation efficiency (EE) and drug loading (DL)

The EE was determined by overspeed centrifugal method. The DMY-PLGA NPs suspension was 100 μL, followed by acetonitrile ultrasonic demulsification for 30 min, centrifugation at 12000 rpm for 10 min, and DMY content was determined by HPLC. The mobile phase consists of methanol and 0.1% phosphoric acid at a ratio of 25:75 (v/v), the stationary phase was C18 column (250 mm×4.6 mm, 5 μm). The injection volume was 10 μL, and the effluent was monitored at 290 nm. The drug load (DL) was determined by freeze-drying method. 1 mL of DMY-PLGA NPs suspension was removed into a weighing bottle, freeze-dried for 48 h and weighed. The freeze-dried powder was dissolved with acetonitrile and the DMY content was determined by HPLC.


EE=WeightofDMYinDMY-PLGANPs/WeightoftheinitialDMY×100%

DL=WeightofDMYinDMY-PLGANPs/Weightofnanoparticles×100%

### 2.4 Cell culture and treatment

H9c2 cardiomyocytes were cultured in high-glucose DMEM medium containing 10% fetal bovine serum and 1% (v/v) penicillin/streptomycin at 37°C and 5% CO_2_. Cells were adherenced to the wall and the growth state of the cells was observed under an inverted microscope. The cells were passed when the growth and fusion of the cells reached 80%-90%.

### 2.5 Establishment of oxidative stress model

The oxidative stress model was established using H_2_O_2_ intervention cells. The cells with good growth were divided into 6 groups: normal group and different H_2_O_2_ concentration groups (200, 400, 600, 800, 1000 μmol·L^-1^). The cells were incubated in the incubator for 4 h according to the corresponding H_2_O_2_ concentration. The survival rate was determined by CCK-8 method to determine the optimal H_2_O_2_ concentration established by the oxidative stress model.

### 2.6 Cytotoxicity assays

Cultured cells with robust growth were seeded into a 96-well plate, with 200 μL of cell suspension per well. The plate was divided into blank, control, different concentrations of DMY, DMY-PLGA NPs, and PLGA NPs (without DMY) groups. After 24 hours of constant-temperature incubation, 100 μL of 10% CCK-8 solution was added to each well. Following a 1 h incubation in the cell culture incubator, absorbance at 450 nm was measured using a microplate reader to calculate cell viability. Each group included 6 parallel wells, and the experiment was repeated three times. Cell viability = (OD_sample_−OD_blank_)/(OD_control_−OD_blank_).

### 2.7 Efficacy test in vitro

The effect of DMY and DMY-PLGA NPs on myocardial cell injury was studied in vitro. Cells were divided into 9 groups, including blank group, control group, model group, DMY and DMY-PLGA NPs concentration groups (12.5, 25, 50 μg·mL^-1^). After incubation at constant temperature for 6, 12, 18 and 24 h, H_2_O_2_ was added, the liquid was sucked up after incubation for 4 h, and 10%CCK-8 solution was added to 100 μL. After incubation in the incubator for 1 h, the absorbance was measured at 450 nm of the enzyme labeled apparatus to calculate the cell survival rate. Six parallel double holes were set in each group, and the experiment was repeated three times. The calculation formula is the same as above.

### 2.8 Determination of LDH、MDA and SOD

Cells were treated in groups and then the supernatant was taken for determination. LDH was determined by colorimetric method, MDA was determined by thiobarbituric acid colorimetric method, and SOD was determined by WST-1 method. The operation was carried out in strict accordance with the kit instructions.

### 2.9 Determination of PGC1α and PPARα protein expression

The protein expression of PGC1α and PPARα was determined using the Western Blotting method. Total protein was extracted with RIPA lysis buffer, and protein quantification was performed using the BCA method. A protein loading volume of 30 μg was applied, followed by SDS-PAGE gel electrophoresis and transfer to a PVDF membrane. The membrane was blocked with 5% skimmed milk for 1 hour at 4°C, then incubated overnight with primary antibodies. After washing three times with TBST, the membrane was incubated with secondary antibodies for 1 hour at 37°C. Following three additional TBST washes, DAB staining was performed, and the images were analyzed using the IBAS 2.5 automated system.

### 2.10 Pharmacokinetics study

#### 2.10.1 Animal grouping, drug administration and sample collection

SD male rats were randomly divided into two groups (n = 6/group): DMY and DMY-PLGA NPs group. After fasting for 12 h, DMY and DMY-PLGA NPs suspension were respectively gavage with the dose of 200 mg·kg^-1^ (measured by DMY amount). After administration, 0.5 mL of blood was collected from jugular vein in anticoagulant tube at 5 min, 10 min, 30 min, 1 h, 1.5 h, 2 h, 3 h, 4 h, 6 h, 7 h, 10 h, 12 h.

#### 2.10.2 Sample pretreatment

The collected whole blood was kept at 4°C for 2 h and 4000 rpm for 15 min. Then 200 μL of upper plasma was taken and added to 10 μL internal standard solution (apigenin, 25 μg·mL^-1^) and 5 μL phosphoric acid. After vortex mixing, the blood was kept at 4000 rpm for 3 min. After extraction with 200 μL ethyl acetate for three times, the supernatant was taken and dried with nitrogen, and redissolved with 100μL methanol. The supernatant was taken at 4000 rpm for 3 min and 80μL was used for detection.

#### 2.10.3 Method establishment

The chromatographic conditions were as follows: Hyperdil BDS C18 column (4.6 mm×200 mm, 5 μm) with acetonitrile as mobile phase A and 0.1% phosphoric acid aqueous solution as mobile phase B, gradient elution: 0∼3 min, 5%-13% A; 3∼8 min, 13∼16%A; 8∼16 min, 16%∼40%A; 16∼25 min, 40%A; 25∼30 min, 40%∼5%A. The flow rate was 1.0 mL·min^-1^, injection volume was 10 μL, and the effluent was monitored at 290 nm. The linear and methodological relationship between blank plasma and DMY were investigated.

### 2.11 Statistical analysis

SPSS 22.0 statistical software was used for statistical analysis, and measurement data were presented as mean ± standard deviation. One-way analysis of variance was used for comparison between groups, and LSD-t test was used for comparison between two groups. Data conversion was performed when variance was inconsistent, and *P* < 0.05 was considered statistically significant.

## 3 Results

### 3.1 Characterization results of DMY-PLGA NPs

**Fig 1:**
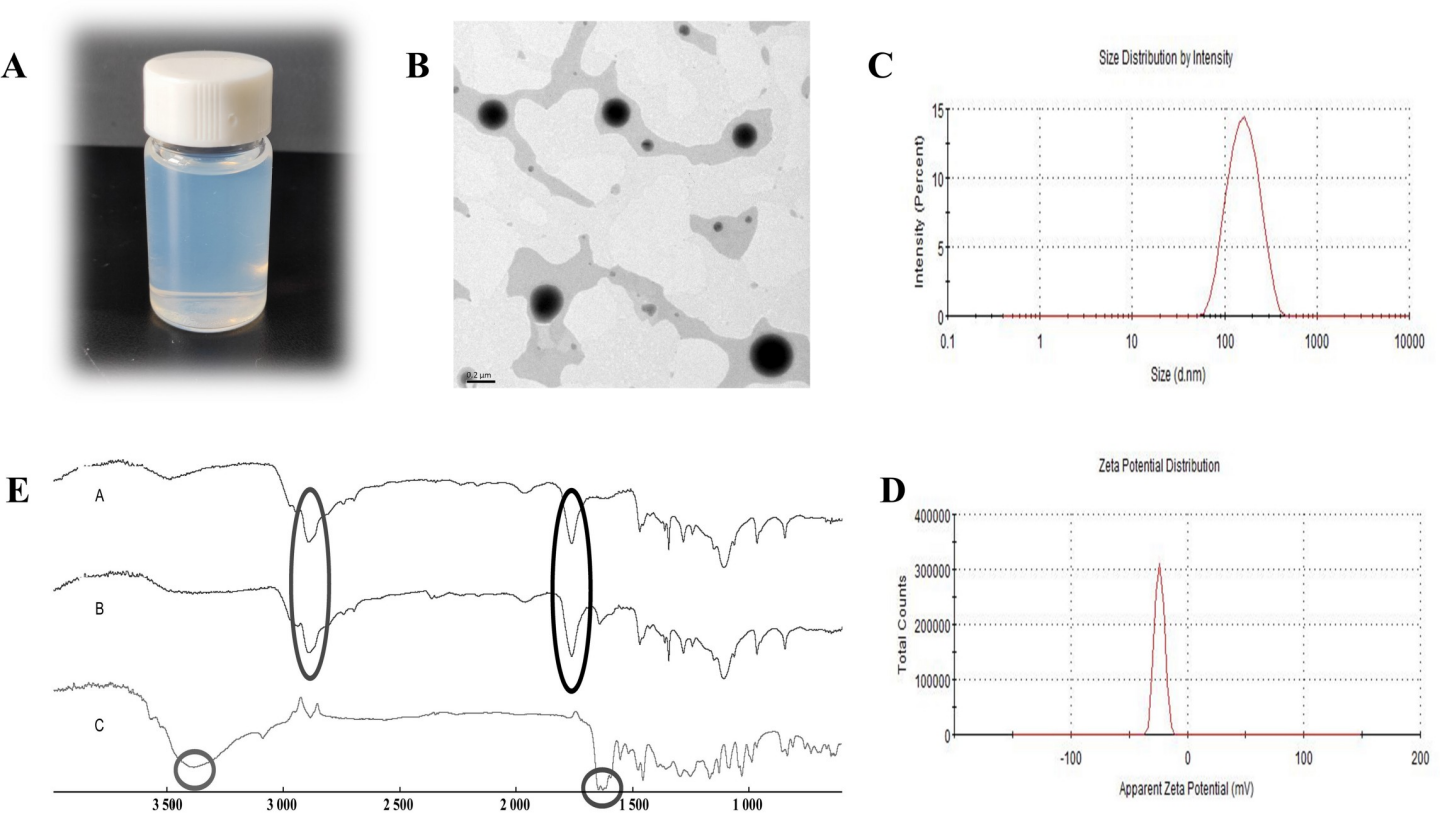
A, Appearance of DMY-PLGA NPs. B, TEM of DMY-PLGA NPs. C, Particle size. D, Potential. E, FTIR results (A is blank nanoparticle, B is DMY-PLGA NPs, C is DMY).

The prepared DMY-PLGA NPs showed a light blue opalescence and a regular spherical shape under TEM. And distribution of particles was relatively uniform and there was no obvious adhesion phenomenon. The average particle size was 147.2 nm, the potential was −33.3 mV, and the PDI was 0.140. The particle size of DMY-PLGA NPs is less than 500 nm, which meets the requirements of the guiding principles of nanopreparations, and the PDI is less than 0.5, which indicates that the distribution is uniform and the dispersion is good. Results of FTIR show that DMY has C = O stretching vibration peak at 1643.70 cm^-1^, and 3382.73 cm^-1^ is -OH stretching vibration peak. The blank nanoparticles have PLGA’s C = O and C-H stretching vibration peaks at 1759.24 cm^-1^ and 2888.66 cm^-1^. The position of the characteristic absorption peaks of drugs and materials in DMY-PLGA NPs remained unchanged, and the kurtosis was slightly smaller, indicating that DMY was successfully encapsulated by PLGA carrier. The EE of DMY-PLGA NPs was 77.41% and the DL was 9.52%. These characterization results of DMY-PLGA NPs are shown in Fig 1.

### 3.2 Establishment of oxidative stress model

**Fig 2:**
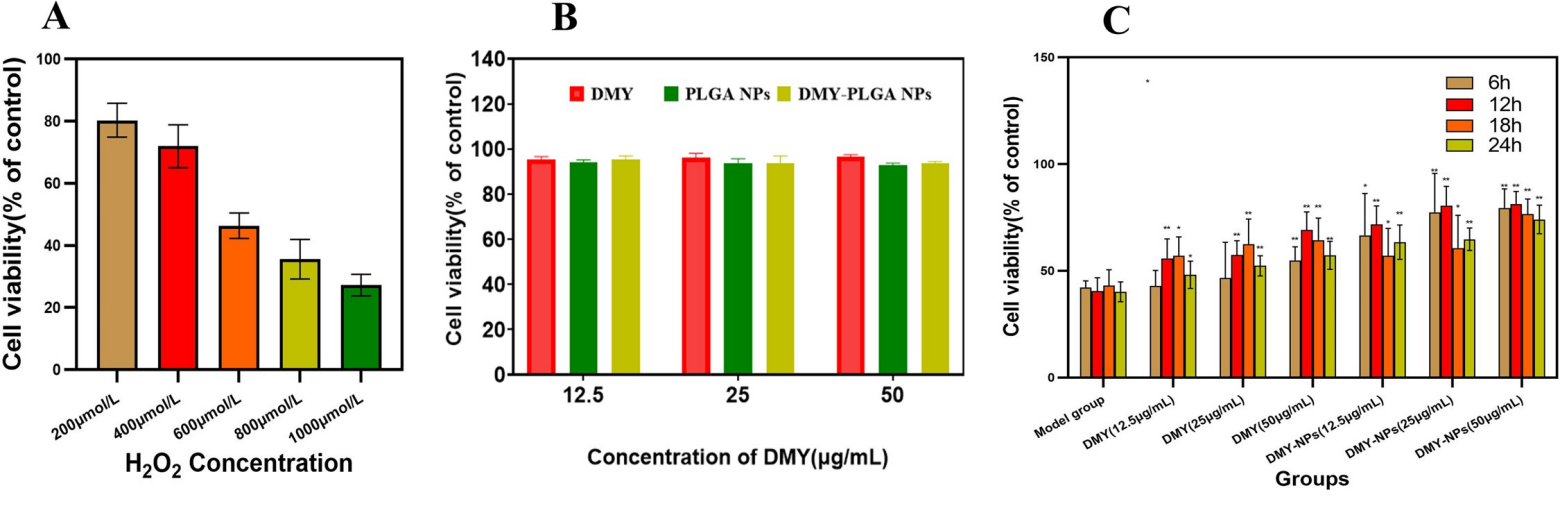
A: Effects of different concentrations of H_2_O_2_ on cell survival rate; B: Results of cytotoxicity test of DMY and DMY-PLGA NPs; C: Results of in vitro efficacy of DMY and DMY-PLGA NPs at different concentrations and different action times. ^*^P < 0.05 vs. model group, ^**^P < 0.01 vs. model group.

With the increase of H_2_O_2_ concentration, the survival rate of H9c2 cardiomyocytes gradually decreased (*P* < 0.01), and the degree of injury was positively correlated with the concentration of H_2_O_2_, as shown in Fig 2A. When H_2_O_2_ concentration is 600 μmol·L^-1^, the cell survival rate is about 45%, while when H_2_O_2_ concentration is 800 μmol·L^-1^, the cell survival rate is reduced to less than 40%, and excessive damage will affect the experimental effect. Therefore, in this study, 600 μmol·L^-1^ H_2_O_2_ concentration was selected to establish the cell damage model.

### 3.3 In vitro cytotoxicity

The control group was normal cells that were not given the drug, and the survival rate was 100%. Compared with the control group, OD values of DMY and DMY-PLGA NPs at different concentrations were not significantly different, and the survival rate of cells after administration was above 98%, as shown in Fig 2B. The results showed that DMY and DMY-PLGA NPs had no obvious toxicity to cardiomyocytes and PLGA material exhibited no significant inhibitory effect on cell viability, which had good biocompatibility.

### 3.4 In vitro efficacy results

The results showed that both DMY and DMY-PLGA NPs could alleviate the damage effect of H_2_O_2_ on cardiomyocytes. After H_2_O_2_ injury, the cell survival rate of the model group was only about 40%, while after DMY-PLGA NPs intervention, the cell survival rate was 70%, as shown in Fig 2C. Moreover, the effect of DMY-PLGA NPs is slightly higher than that of DMY, indicating that DMY can prolong its action time in vivo and enhance its drug efficacy after being prepared into nanoparticles. In addition, the protective effect of different administration times on cardiomyocytes was also different. The survival rate of cardiomyocytes was the highest at 12 h of administration, and it was not significantly increased after 12 h, which may be due to the gradual degradation of PLGA, which led to the slow release of DMY, and the gradual reduction of DMY concentration due to the beginning of metabolism of DMY. Therefore, 12 h was selected as the final administration time for follow-up experiments.

### 3.5 Changes of LDH, MDA and SOD contents

**Fig 3:**
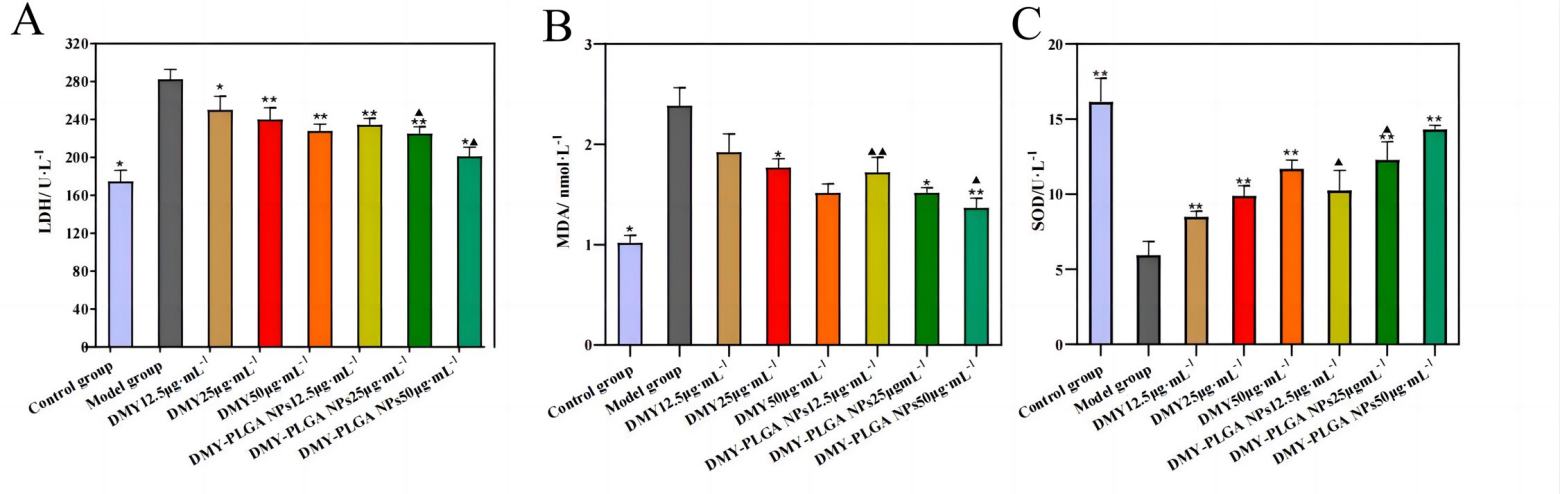
Effects of DMY and DMY-PLGA NPs on changes in LDH, MDA and SOD contents. A: LDH; B: MDA; C: SOD. ^*^P < 0.05 vs. model group, ^**^P < 0.01 vs. model group. ^▲^*P* < 0.05 vs. DMY group, ^▲▲^*P* < 0.01 vs. DMY group.

Compared with normal cells, the contents of LDH, MDA increased and SOD decreased in the model group, indicating that the oxidative stress model was successfully established. After treating cells with DMY and DMY-PLGA NPs for 12 h, LDH and MDA levels were significantly decreased and SOD level was increased, indicating that both DMY and DMY-PLGA NPs could alleviate oxidative damage of cells, and DMY-PLGA NPs had better effects than DMY, as shown in Fig 3.

### 3.6 Expression of PGC1α and PPARα proteins

**Fig 4:**
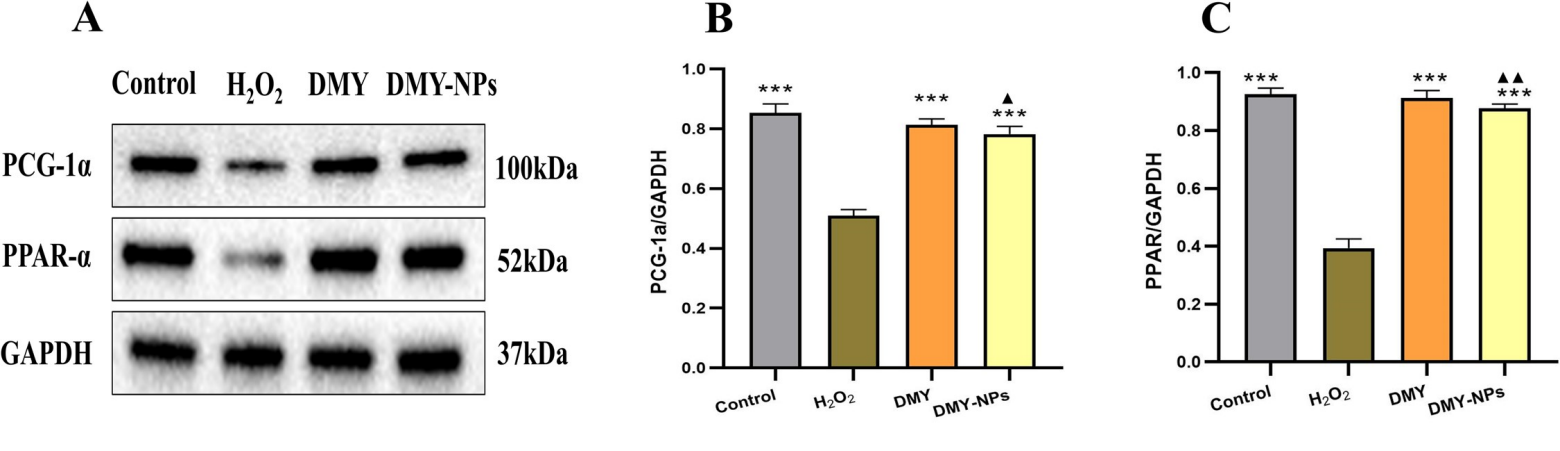
A: expression of PGC1α and PPARα regulated by DMY and DMY-PLGA NPs; B: quantitative figure of PGC1α; C: quantitative figure of PPARα. ^**^P < 0.01 vs. model group, ^***^P < 0.001 vs. model group. ^▲^*P* < 0.05 vs. DMY group, ^▲▲^*P* < 0.01 vs. DMY group.

Compared with the control group, the protein contents of PGC1α and PPARα in H_2_O_2_ model group were significantly reduced, and the protein expression was up-regulated by DMY and DMY-PLGA NPs. Besides, the regulation effect of DMY-PLGA NPs was slightly higher than that of DMY. The results showed that both DMY and DMY-PLGA NPs could up-regulate the expression of PGC1α and PPARα to reduce oxidative damage of cardiomyocytes induced by H_2_O_2_, as shown in Fig 4. Raw images of proteins are shown in S1 Raw images.

### 3.7 Methodological study of pharmacokinetics

**Fig 5:**
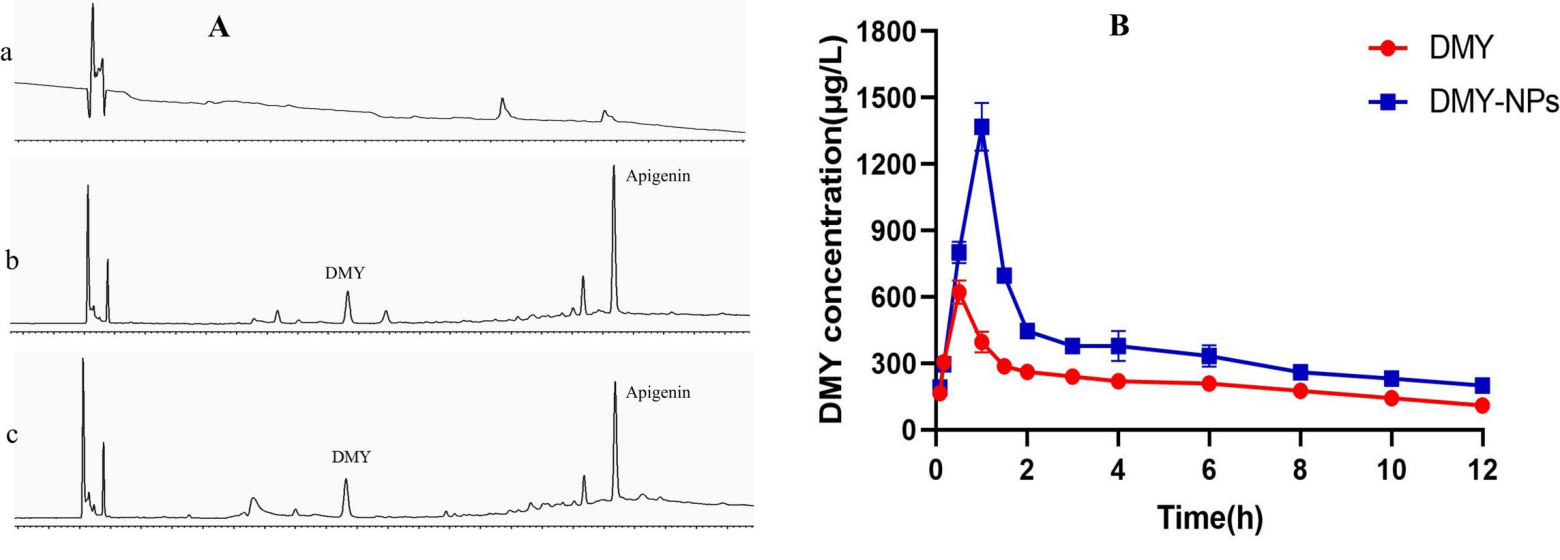
A: Specific HPLC diagram of DMY and internal standard solution (a: blank plasma b: containing control plasma c: plasma sample); B: DMY and DMY-PLGA NPs drug-time curves.

The linear regression equation was established with the ratio of DMY to internal standard peak area (Y) and solution mass concentration (X): Y = 0.0003X−0.001 (*r* = 0.999), and the linear relationship was good in the range of 20∼100 μg·L^-1^. The RSD of high, medium and low mass concentration plasma (80, 250, 800 μg·L^-1^, measured by DMY amount) was 3.89%, 8.36% and 10.03%, the RSD of stability was 5.93%, 3.73% and 10.64%, and the RSD of instrument precision was 5.97%. The recoveries were 88.18%∼94.26%. According to the results of comprehensive methodological investigation, the established HPLC method and plasma treatment method is suitable for the analysis of DMY, and the HPLC specific chromatogram is shown in Fig 5A. The original pharmacokinetic data are shown in S1 Data.

### 3.8 Results of pharmacokinetics

The blood concentration-time curve is shown in Fig 5B, and the parameters are shown in Table 1. Plasma DMY reached a peak of 621.41 μg·L^-1^ after 0.5 h of DMY administration, while DMY-PLGA NPs reached a peak concentration of 1369.42 μg·L^-1^ at 1 h. Compared with DMY, the AUC0∼∞ of DMY-PLGA NPs was increased, which was 2.04 times of DMY, and the half-life was 1.46 times of DMY. The results showed that the degradation ability of DMY was decreased after PLGA inclusion, and it was not easy to be cleared by the body and the cycle time was prolonged.

**Table 1:** Pharmacokinetic parameters (^*^P < 0.05 vs. DMY, ^**^P < 0.01 vs. DMY).

Parameters	DMY	DMY-PLGA NPs
C_max_ (μg/L)	621.41±52.31	1369.42±108.02^**^
T_max_ (h)	0.5	1
AUC_0→t_ (μg /L)·h	2610.32±106.34	4586.00±126.80^**^
AUC_0→∞_ (μg /L)·h	3711.65±241.8	7557.51±1282.01^**^
CL (L/h/kg)	54.08±3.52	26.976±3.63^**^
T_1/2_ (h)	6.943±1.20	10.121±3.00^*^

## 4 Discussion

DMY is an active substance that has significant pharmacological effects on many organs of the body. For example, DMY can effectively reduce the size of hematoma and cell apoptosis in brain tissue, increase the expression of LCN2, and inhibit ferroptosis caused by SLC3A2 to alleviate cerebral hemorrhage [18]. In addition, when DMY was used to interfere with fibrotic lung injury induced by methotrexate, it was found through lung histopathological examination that DMY could reduce the levels of NF-κB, IL-1β and TGF-β1 to inhibit lung inflammation and pulmonary fibrosis [19]. DMY also has a therapeutic effect on kidney lesions. After acute kidney injury induced by lipopolysaccharide was treated with DMY, the lesion site was improved, it may alleviate acute kidney injury by regulating the effect of miR-199b-3p on Nrf2 pathway [20]. In this study, we paid more attention to the therapeutic effect of DMY on cardiovascular diseases, especially myocardial ischemia. Studies have shown [6, 21] that DMY can up-regulate Sirt3 to restore mitochondrial function, regulate NLRP-3, NF-κB and other inflammatory factors, thereby reducing the infarct size and restoring cardiac function. Another experiment [22] studied the protective effect of DMY on myocaromyocytes by establishing in vitro cardiomyocyte H/R model and in vivo I/R injury model, and the results showed that DMY had a good protective effect on myocardial injury, the mechanism of which may be related to the activation of PI3K/Akt and HIF-1α signaling pathway.

Targeting the significant protective and repair effects of DMY on myocardium is crucial for its development and utilization to maximize its efficacy. In recent years, there has been a large amount of research work to develop DMY into different dosage forms or delivery systems to evaluate its efficacy. Some people [23] made drug eutectic with DMY and pentoxifylline, and the characterization results showed that the solubility of DMY was significantly improved and the simultaneous release of the drug was realized. In addition, by making DMY into liposomes and establishing C57BL/6 mouse inflammation model, in vitro flow results showed that DMY liposomes could induce macrophage polarization to effectively alleviate inflammation [24]. In another study [25], DMY was assembled into micelles, and compared with DMY, the solubility of DMY micelles increased by 25 times, and the bioavailability was also increased to 205% through intestinal perfusion. In our study, PLGA was used as the carrier and DMY was prepared into nanoparticles by emulsification method, in which poloxam was added as a co-solvent. The prepared DMY-PLGA NPs was relatively clear and visible to the naked eye with a light blue opalescence. The characterization results showed that the particle size was 147.2 nm, the potential was −33.3mV, and the PDI was 0.140. TEM showed that the DMY was round and evenly distributed, and FTIR also confirmed that DMY was successfully encapsulated in PLGA. Therefore, a stable and reliable DMY-PLGA NPs was successfully prepared in this study.

The development process of MIRI is closely related to mitochondrial energy metabolism and oxidative stress [4, 26], and mitochondrial energy metabolism can often affect the occurrence of oxidative stress, indicating that oxidative stress plays a huge role in improving MIRI. H_2_O_2_ is a common oxygen free radical molecule. As an active oxygen species, it easily reacts with iron ions in cells through cell membranes, resulting in abnormal oxygen levels in cells. Therefore, in this study, the oxidative stress model of H9c2 cardiomyocytes was constructed by H_2_O_2_ to explore the therapeutic effect of DMY-PLGA NPs on MIRI.

The results of in vitro experiments showed that both DMY and DMY-PLGA NPs had protective effects on cardiomyocytes injured by H_2_O_2_. After treatment with DMY and DMY-PLGA NPs, the levels of LDH and MDA in cells were significantly decreased, SOD level was increased, and PGC1α and PPARα protein expressions were increased. The effect of DMY-PLGA NPs was stronger than that of DMY. LDH, a class of NAD dependent kinases, is a crucial oxidoreductase in the glycolytic pathway in vivo [27], and therefore is often used as a test index for myocardial diseases. MDA is one of the commonly used indexes to measure the degree of oxidative stress. In vivo, MDA is the final product of oxidation reaction of free radicals. It contains relatively high cytotoxicity, which is easy to cause the destruction and dysfunction of macromolecular structures such as proteins in cells, resulting in oxidative damage of the body [28, 29]. An antioxidant metalloenzyme existing in SOD organism, it can catalyze superoxide anion radical disproportionation, and plays a crucial role in the balance between oxidation and antioxidant in the body. In the treatment of cardiovascular diseases, SOD can remove excessive oxygen free radicals in the lesion site, regulate blood lipids and hemorheology, and play a role in protecting the heart muscle [30, 31]. As a widely expressed transcription factor, PGC1α protein is often involved in lipid metabolism, mitochondrial biosynthesis and oxidative stress expression [32]. Studies have shown that the SIRT1-mediated Nrf2 pathway in chondrocytes [33] can inhibit PGC1α expression, leading to mitochondrial dysfunction and thus oxidative stress. PPARα has a similar functional role to PGC1α, but it can regulate the fusion of autophagosome and lysosome [34] and reduce mitochondrial damage and oxidative stress. DMY and DMY-PLGA NPs protect cardiomyocytes by regulating the content of these markers and protein pathways.

In vivo pharmacokinetic results showed that after oral administration of DMY and DMY-PLGA NPs, the concentration of DMY-PLGA NPs in vivo was accumulated, and the half-life of DMY-PLGA NPS was also prolonged. This is due to the role of PLGA, when DMY is wrapped by PLGA, it is like wearing a "protective film", which can reduce the cell’s clearance of DMY and extend the action time in the body. Therefore, through characterization and in vitro and in vivo evaluation, we successfully prepared DMY-PLGA NPs, which can effectively relieve oxidative stress caused by H_2_O_2_, protect H9c2 cardiomyocyte damage, and improve oral bioavailability, providing a certain reference for the treatment of MIRI.

## 5 Conclusions

In this study, we have successfully prepared the DMY-PLGA NPs, and the characterization results show that DMY-PLGA NPs are round and stable. The results of in vitro experiments showed that both DMY and DMY-PLGA NPs could alleviate the oxidative damage of H9c2 cardiomyocytes induced by H_2_O_2_ by decreasing the levels of LDH and MDA and increasing the levels of SOD. The mechanism of action may be related to upregulated PGC1α and PPARα proteins. Pharmacokinetic studies also have shown that DMY-PLGA NPs can improve oral bioavailability compared to DMY. However, this study also has some shortcomings. The efficacy of DMY-PLGA NPs still needs to be proved through a series of in vivo experiments, and how to achieve targeted drug delivery of DMY-PLGA NPs is also a problem that we will further solve.

## Supporting information

S1 Raw images Raw images of proteins. (DOCX)

S1 Data Original pharmacokinetic data. (XLSX)

S1 Graphical abstract (TIF)
