# Supplementary figures and images for: Protective effect and pharmacokinetics of dihydromyricetin nanoparticles on oxidative damage of myocardium
Source: PLoS One. 2024 Apr 16;19(4):e0301036. doi: 10.1371/journal.pone.0301036 (PMC11020404; doi:10.1371/journal.pone.0301036)

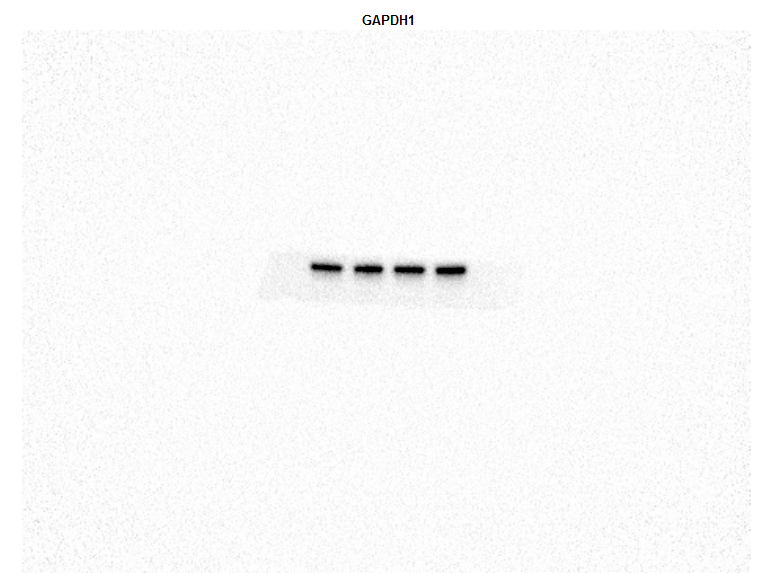


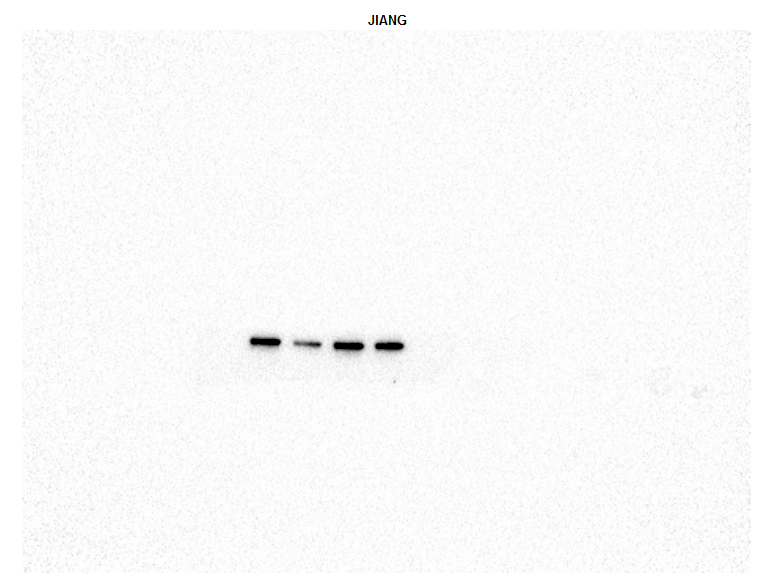

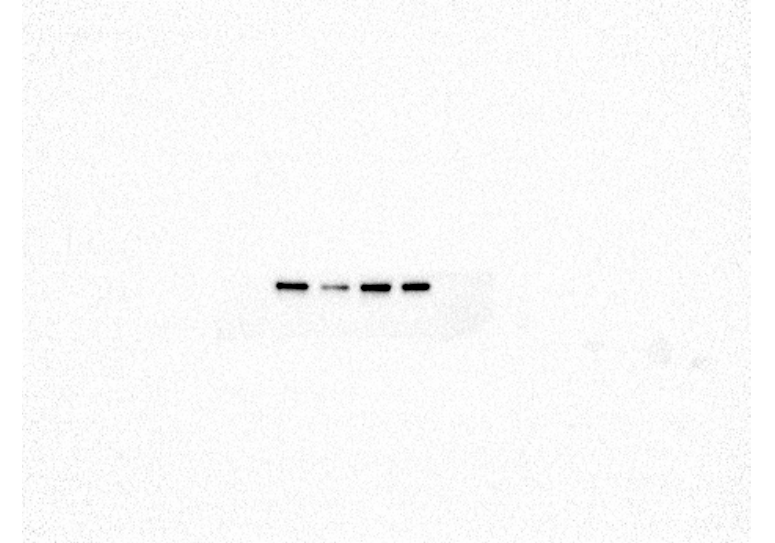


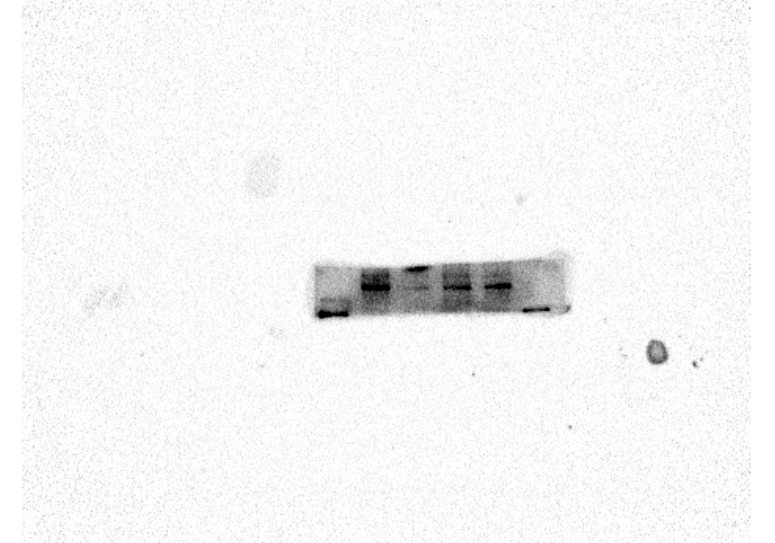

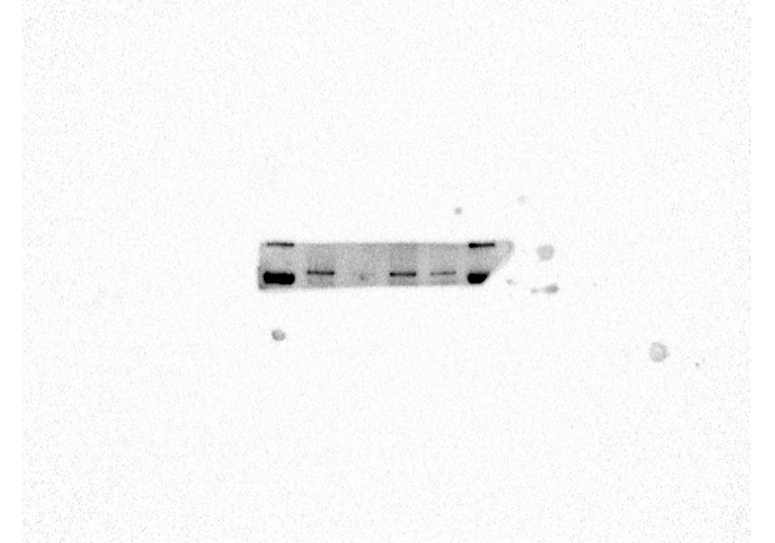

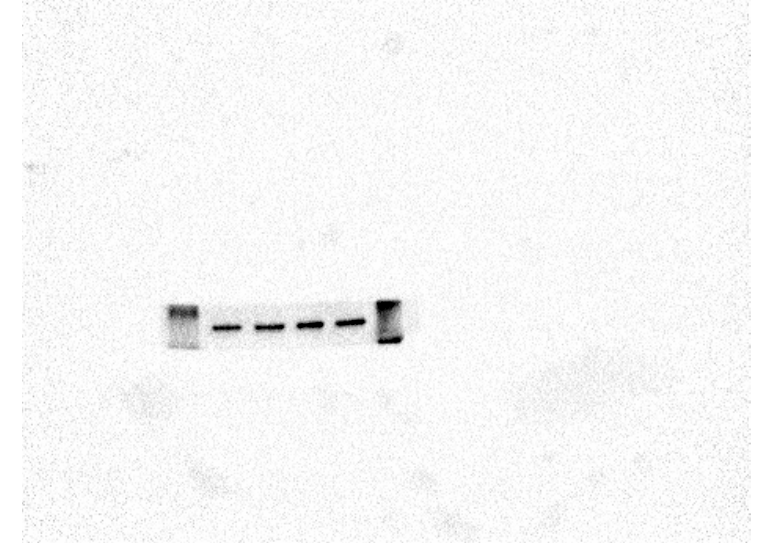

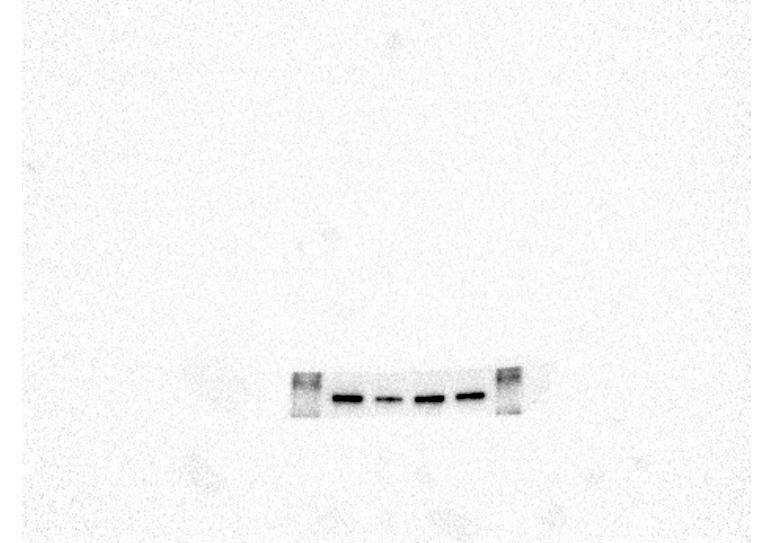

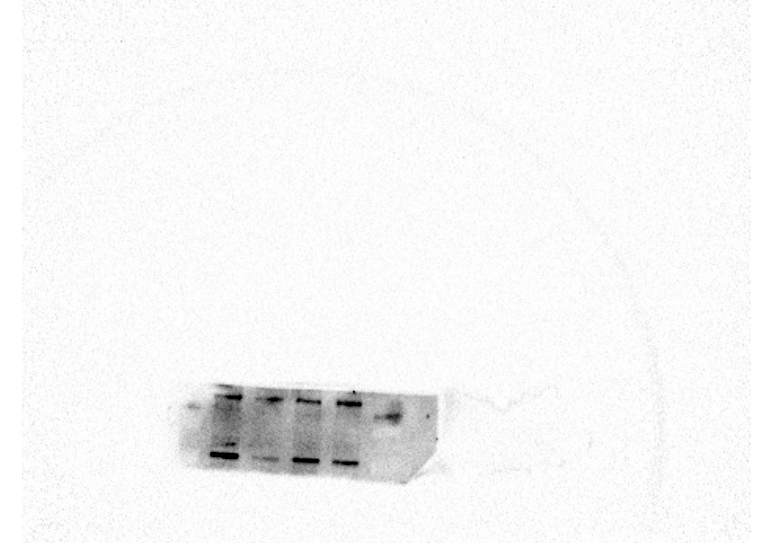

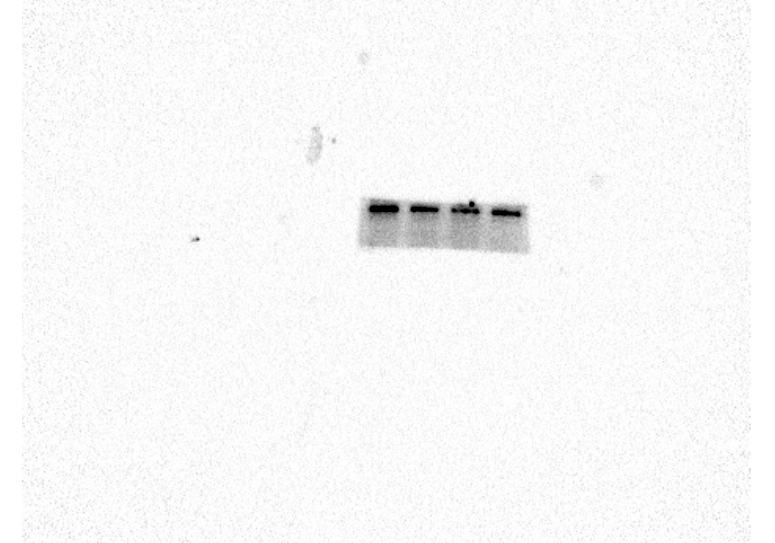

Supplement: S1 Raw images — (DOCX) [file pone.0301036.s001.docx]

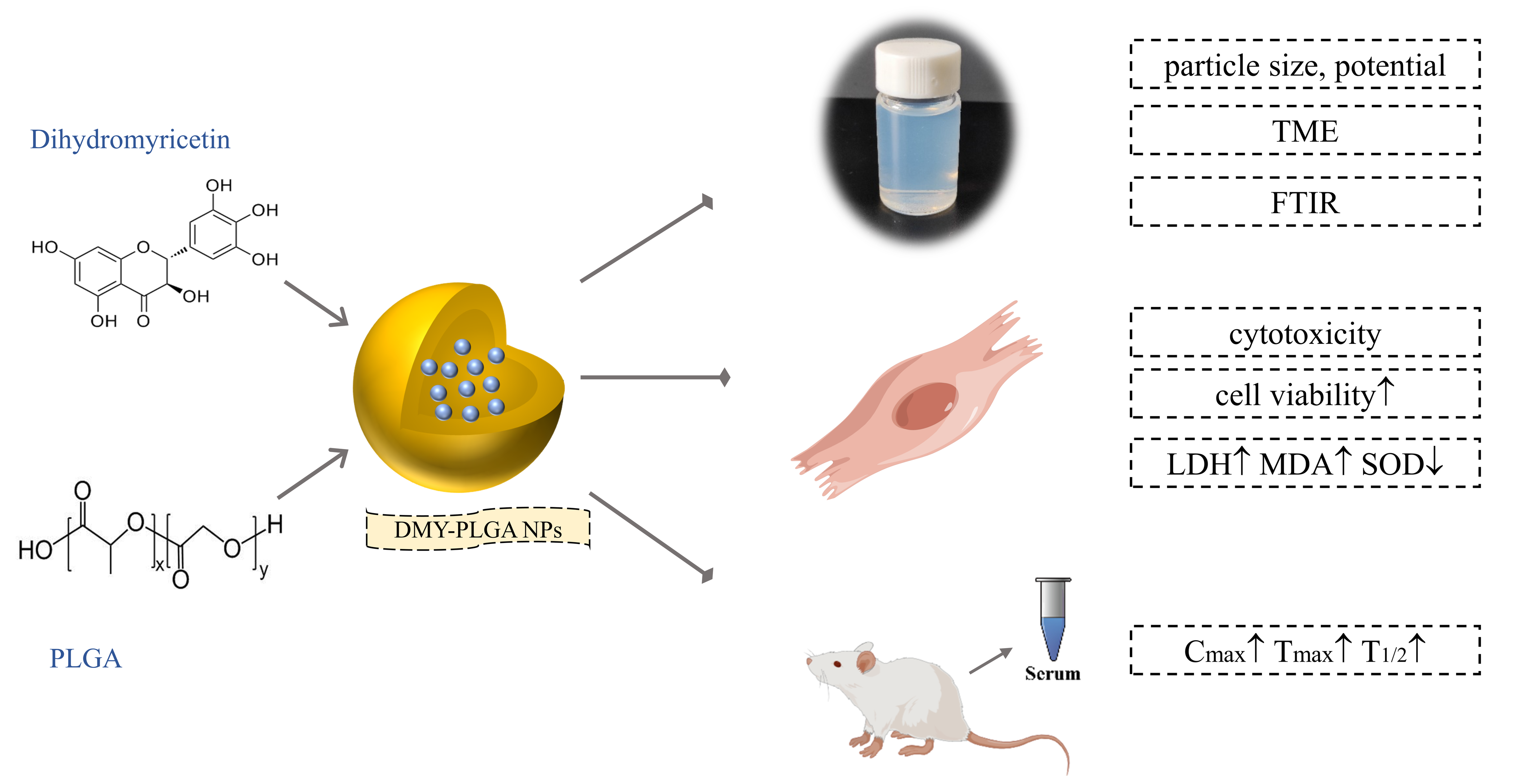

Supplement: S1 Graphical abstract — (TIF) [file pone.0301036.s003.tif]
